# Triggering of Protection Mechanism against *Phoneutria nigriventer* Spider Venom in the Brain

**DOI:** 10.1371/journal.pone.0107292

**Published:** 2014-09-11

**Authors:** Catarina Rapôso, Paulo Alexandre Miranda Odorissi, Stefania Fioravanti Savioli, Rafaela Chitarra Rodrigues Hell, Gustavo Ferreira Simões, Roberta R. Ruela-de-Sousa, Alexandre Leite Rodrigues de Oliveira, Maria Alice da Cruz-Höfling

**Affiliations:** 1 Department of Biochemistry and Tissue Biology, State University of Campinas- Unicamp, Campinas, São Paulo, Brazil; 2 Department of Functional and Structural Biology, State University of Campinas- Unicamp, Campinas, São Paulo, Brazil; Emory University School of Medicine, United States of America

## Abstract

Severe accidents caused by the “armed” spider *Phoneutria nigriventer* cause neurotoxic manifestations in victims. In experiments with rats, *P. nigriventer* venom (PNV) temporarily disrupts the properties of the BBB by affecting both the transcellular and the paracellular route. However, it is unclear how cells and/or proteins participate in the transient opening of the BBB. The present study demonstrates that PNV is a substrate for the multidrug resistance protein-1 (MRP1) in cultured astrocyte and endothelial cells (HUVEC) and increases *mrp1* and *cx43* and down-regulates *glut1* mRNA transcripts in cultured astrocytes. The inhibition of nNOS by 7-nitroindazole suggests that NO derived from nNOS mediates some of these effects by either accentuating or opposing the effects of PNV. *In vivo*, MRP1, GLUT1 and Cx43 protein expression is increased differentially in the hippocampus and cerebellum, indicating region-related modulation of effects. PNV contains a plethora of Ca^2+^, K^+^ and Na^+^ channel-acting neurotoxins that interfere with glutamate handling. It is suggested that the findings of the present study are the result of a complex interaction of signaling pathways, one of which is the NO, which regulates BBB-associated proteins in response to PNV interference on ions physiology. The present study provides additional insight into PNV-induced BBB dysfunction and shows that a protective mechanism is activated against the venom. The data shows that PNV has qualities for potential use in drug permeability studies across the BBB.

## Introduction

Accidents involving the “armed” spider *Phoneutria nigriventer* can cause neurotoxic manifestations, which in severe cases may include convulsion [1]. In experiments involving rats, *P. nigriventer* venom (PNV) impairs the BBB by increasing endothelial transcellular vesicular transport mediated by microtubules [2] and by down-regulation of inter-endothelial junctional proteins [3], [4]. In astrocytes, PNV induces swelling of the perivascular end-feet [5], increases cytoskeletal GFAP and S-100 calcium metabolism-associated protein expression [6], and upregulates aquaporin-4 [7], a water channel-forming protein involved in edema formation and resolution. In neurons, PNV causes cFOS induction [3] and upregulation of VEGF and Flt-1 and Flk-1 receptors [8], [9]. Increased understanding of the mechanisms involved in BBB dysfunction caused by PNV is beneficial from a medical perspective.

In the present study, MRP1 efflux pump activity was examined in cultured astrocytes and endothelial cells (HUVEC lineage) incubated with PNV. The effect of PNV on the mRNA transcripts of *mrp1*, *glut1* and *cx43* in astrocytes and the mediation of NO in the same was also analyzed. *In vivo*, the expression of these proteins was examined in the hippocampus and cerebellum of rats at different time-points following PNV exposure. The present study is of interest as investigations into the effect of animal venom on the BBB are rare. The use of PNV as a pharmacological tool to manipulate BBB can provide new insights on the changes that occur at the blood-brain interface. Spider venoms represent a rich mixture of proteins, peptides, neurotransmitters and small molecules with specific, highly potent effects on the nervous system of their prey or predators [10]. *P. nigriventer* venom contains Ca^2+^, K^+^ and Na^+^ channel-acting neurotoxins that affect neurotransmitter release and uptake [11]. Therefore its instrumental use for pharmacological characterization of proteins associated with the functioning of the BBB could be important for evaluating the possible therapeutic potential of PNV in neurochemical disturbances [10], [12], [13]. A positive factor is that severe envenomation by *P. nigriventer* occurs in less than 0.5% of accidents involving humans [1], and that in experimental cases the effects of PNV are resolved in a few hours. Thus it is important to further investigate the effect of PNV-induced BBB opening, taking into consideration the pharmacological usefulness of the venom for drug permeability studies across the BBB. The study also contributes to a greater understanding of the envenomation profile of accidents involving *P. nigriventer*.

## Material and Methods

### Animals

*In vitro* assays used newborn Wistar rats (*Rattus norvegicus*, 0–2 days old) for harvesting astrocytes and *in vivo* assays used male Wistar rats (250–300 g), all obtained from an established colony maintained by the Multidisciplinary Center for Biological Investigation (CEMIB) at UNICAMP. Animals were kept in a 12/12 hour-light/dark cycle with food and water supplied *ad libitum*. The experiments were approved by the Institutional Committee for Ethics in Animal Use (CEUA, protocol no. 1429-1) and followed the Brazilian College for Animal Experimentation (COBEA) guidelines.

### Purified astrocyte primary cultures and HUVEC cells

A primary culture of cerebral cortical-derived astrocytes was purified from 0–2 day-old newborn Wistar rats as previously described [4], [14]. Human umbilical endothelial cell lines (HUVEC, ATCC #CRL-1730, Manassas, VA, USA) were cultured in DMEM containing 10% fetal bovine serum (FBS), supplemented with 100 U/ml penicillin, 100 mg/ml streptomycin, and propagated at 37°C in a 5% CO_2_ humidified atmosphere. Cells were seeded onto 24-well cell culture plates (10^4^ cells/well).

### In vitro MRP1 efflux activity after treatment with PNV or PNV plus nNOS blocker

Measurement of MRP1 activity in astrocytes and HUVEC was performed using a fluorescein substrate. Astrocytes or HUVEC were placed in a 6-well plate and treated with DMEM (control), PNV (14.6 µg/ml), only 7NI (control) or PNV plus 7NI (1 mg/ml) for 5 h. In the last two hours of treatment, a fluorescein substrate (100 µM) was added to the cells (uptake phase). The cells were then washed with PBS and incubated for 15 min in a completely fresh medium, without fluorescein, to allow fluorescein efflux by MRP1 (efflux phase). To evaluate the effect of PNV, PNV plus 7NI, and 7NI directly on MRP1 activity, these were added only during the efflux phase, for 15 min. Fluorescein fluorescence was assessed by flow cytometry analysis within the live-gate, using FACScalibur (Becton and Dickinson - BD, Franklin Lakes, NJ, USA). Data was analyzed using Cell Quest Pro software (Becton and Dickinson – BD). The values correspond to the maximum fluorescence resulting from fluorescein uptake and are expressed in Relative Fluorescence Units (RFU). The values were obtained through three sets of experiments.

### In vitro mrp1, glut1 and cx43 mRNAs expression in astrocytes treated with PNV or PNV plus nNOS blocker

Cultured astrocytes were treated with 1) DMEM (control); 2) 14.6 µg/ml of PNV freshly-diluted in DMEM [2]; 3) 7NI (control); 4) PNV plus selective nNOS blocker, 7-nitroindazole (7NI, 1 mg/ml) [15] for 2 h at 37°C. The mRNAs of the three proteins were measured. Then, astrocytes (∼1×10^6^ cells) were trypsinized, washed and the total RNA was extracted using a commercial extraction kit in accordance with the manufacturer's instructions (Absolutely RNA Miniprep-GE). The concentration of isolated RNA samples was assessed by molecular absorption spectroscopy in the UV region, at 260 nm, and purity was assessed by calculating the ratio between absorbance at 260/280 nm. The reverse transcription from each sample (1 µg of total RNA) was performed with AffinityScript QPCR cDNA Synthesis Kit (Agilent Technologies, Santa Clara, CA, USA) in accordance with manufacturer's instructions. Reverse transcription was carried out at 25°C for 5 min, and the products were then extended at 42°C for 30 min to allow cDNA synthesis. Finally, the reaction was terminated by heating at 95°C for 5 min. Samples without reverse transcriptase were processed in parallel and served as negative controls. The effect of PNV on the expression of several genes encoding MRP1, GLUT1 and Cx43 proteins was investigated by qRT-PCR using the Brilliant II SYBR Green QPCR Master Mix on Mx3005P QPCR Systems (Agilent Technologies). Thermal cycling conditions were as follows: 10 min at 95°C followed by 40 amplification cycles at 95°C for 30 s, 55°C for one min and 72°C for one min. Depending on the type of primer, each reaction was performed using 1.0, 0.5 or 0.25 µM of forward and reverse primer and 100 ng of cDNA template in a final reaction volume of 25 µl. Melting curve analysis was performed at the end of the PCR to verify the identity of the products. The denaturation temperature was determined at the end of the amplification program, in a dissociation protocol that consisted of gradual 60°C to 95°C temperature increase at 0.5°C steps. The initiators were delineated based on the mRNA sequence described in Table 1. Additionally, whenever possible, PCR primers were designed to span adjacent exons in order to prevent amplification of the intron-containing genomic DNA. An efficiency curve was carried out for each primer using the serial dilution method. All quantifications were normalized to the housekeeping gene GAPDH and evaluated based on the control group from the Ct of these groups for each tested gene. A non-template control with non-genetic material was included to eliminate contamination or nonspecific reactions. Each sample was tested in duplicate and then used for the analysis of the relative transcription data using the 2^−ΔΔCT^ method [16], taking into account the efficiency of each primer [17].

**Table 1:** Sequences of primers of gapdh, cx43, mrp1 and glut1.

Primer	Sequence	NCBI Reference number
gapdh	5′ GGCTCTCTGCTCCTCCCTGTTCT 3′	NM_017008.3
	5′ CCGTTCACACCGACCTTCACCATC 3′	
cx43	5′ CTTCAGCCTCCAAGGAGTTCCACCA 3′	NM_012567.2
	5′ CAGTCACCCATGTCTGGGCACC 3′	
mrp1	5′ GATGGCTCCGATCCGCTCTGG 3′	NM_022281.2
	5′ GGCACCCATGTGAGGACCGTATTCT 3′	
glut1	5′ GTTTCACAGCCCGCACAGCTTGA 3′	NM_138827.1
	5′ GCCCCTCCCACGGCCAACATA 3′	

### In vivo assays: Western blotting and immunofluorescence of MRP1, GLUT1 and Cx43

The animals (250–300 g) were divided into two groups. One group received a single intravenous (i.v.) injection of PNV (850 µg/kg in 0.5 ml) [5] in the tail vein, while the control (sham) group received the same volume of 0.9% sterile saline solution. At 30±15 min, 2 h and 5 h post-injection (n = 5/time interval) the animals were killed with a 3∶1 i.p. overdose of ketamine chloride (Dopalen; 100 mg/kg) and xylazine chloride (Anasedan; 10 mg/kg) anesthetics (Vetbrands, Jacarei, SP, Brazil). This time-window refers to the period of acute intoxication of the rats and their ongoing clinical recovery from a toxic condition [18].

#### *Western Blotting (WB)*

Deeply-anesthetized (CO_2_ inhalation) animals were euthanized by decapitation. Cerebella and hippocampi were quickly dissected and homogenized in lysis buffer (10 mM EDTA, 2 mM PMSF, 100 mM NaF, 10 mM sodium pyrophosphate, 10 mM NaVO_4_, 10 mg of aprotinin/ml and 100 mM Tris, pH 7.4). Because control animals were alive and did not show signs of clinical impairment, their hippocampi and cerebella (from 30±15 min, 2 h or 5 h time-points) were mixed and homogenized to form a control pool (n = 15). The expression of proteins of the control pool was compared with the PNV-30 ±15 min, PNV-2 h or PNV-5 h (n = 5 per time) group. Homogenates were processed and underwent immunoblotting as described [6]. Briefly: after centrifugation (3000 g/10 min), the supernatant was collected and stored at −70°C. The proteins (50 µg) were separated on sodium dodecyl sulfate-polyacrylamide and electrophoretically transferred onto the nitrocellulose membrane (BioRad Laboratories, ref. 162-0115). After overnight blocking (5% non-fat milk) the membranes were incubated at room temperature for 4 h with primary antibodies described in *Imunofluorescence* sub-section (MRP1 – 1∶200; GLUT1 – 1∶400; Cx43 – 1∶600 dilutions), followed by secondary horseradish peroxidase-antibodies (1∶1000, Sigma). The blots were revealed using a chemiluminescence reagent (Super Signal, Pierce) and X-ray film (Fuji Medical, Kodak, Ref Z358487-50EA). Each band was quantified by densitometry using the IMAGEJ software (version 1.33u, NIH, USA). For each protein studied the results were confirmed in three sets of experiments. The blots were then stripped and reprobed for anti-β-actin analysis (1∶250, Sigma) as a loading control for the other protein blots. Negative control was provided by omitting the primary antibody.

#### *Immunofluorescence (IF)*

Rats were deeply anesthetized and then sequentially perfused transcardially with 150 ml of physiologic solution followed by fixative ((250 ml of ice-cold 0.1 M phosphate-buffered saline (PBS) containing 4% paraformaldehyde, pH 7.4)). The brains were dissected, frozen in liquid N_2_ and cryoprotected with 15% and 30% sucrose solution (24 hours each). Cryosections (12 µm thick) were mounted on silane-coated glass slides, air dried and then permeabilized with 0.1% Triton X-100 for 10 min at room temperature (RT) (only for GLUT1, before Triton X-100, the slices were incubated with ethanol, followed by methanol, for 10 min each, at −20°C), followed by 0.1% Tween 20 in TBS containing 5% non-fat milk at RT for 1 h. Subsequently, sections were reacted with anti-MRP1 (Zymed, 187246 - 1∶100), anti-GLUT1 (Alpha Diagnostic, GT11-S - 1∶100), anti-Cx43 (Sigma, C6219 – 1∶500) and GFAP (Dako Cytomation, CA, USA, ZO 334 – 1∶100) at 4°C (for GLUT1 only the incubation lasted 2 h in RT), and with FITC- or TRITC-conjugated polyclonal secondary antibody (Anti-rabbit IgG TRITC conjugate – T5268 – 1∶1500; Anti-mouse IgG FITC conjugate – F6257 – 1∶1500, Sigma) at RT for 1 h in the dark. Six images/animal ( = 30 images/time) were captured at random using a fluorescence BX51TF microscope (Olympus Optical C. Ltd., Tokyo, Japan) equipped with Image-Pro Plus 6.0 image analyzer software (Media Cybernetics Inc., USA). Quantification of the protein expression was performed with enhanced contrast and the density slicing feature of IMAGEJ software (version 1.33u, NIH, USA), which measures the intensity of immunofluorescence. Four sections per animal at each of the time-points (n = 5 animals per time  = 20 sections/time) for the control and envenomed groups, were immunolabeled. The integrated density of pixels was measured by the total area (0.35 mm^2^) of captured images by C.R. and P.A.M.O. (double blinded measure).

### Statistical analysis

The results were expressed as mean ± S.E.M. or mean ± S.D.M. All the numerical data were analyzed using the GraphPad Prism software package. One-way analysis of variance (ANOVA) followed by the Tukey-Kramer *post*-test was used to compare data from the control and PNV-treated samples. Statistical significance was set at *p*<0.05. Unpaired *t*-Student was used to compare each treatment with control.

## Results

### In vitro experiments: MRP1 activity

#### *PNV-treated astrocytes*

**Figure 1:**
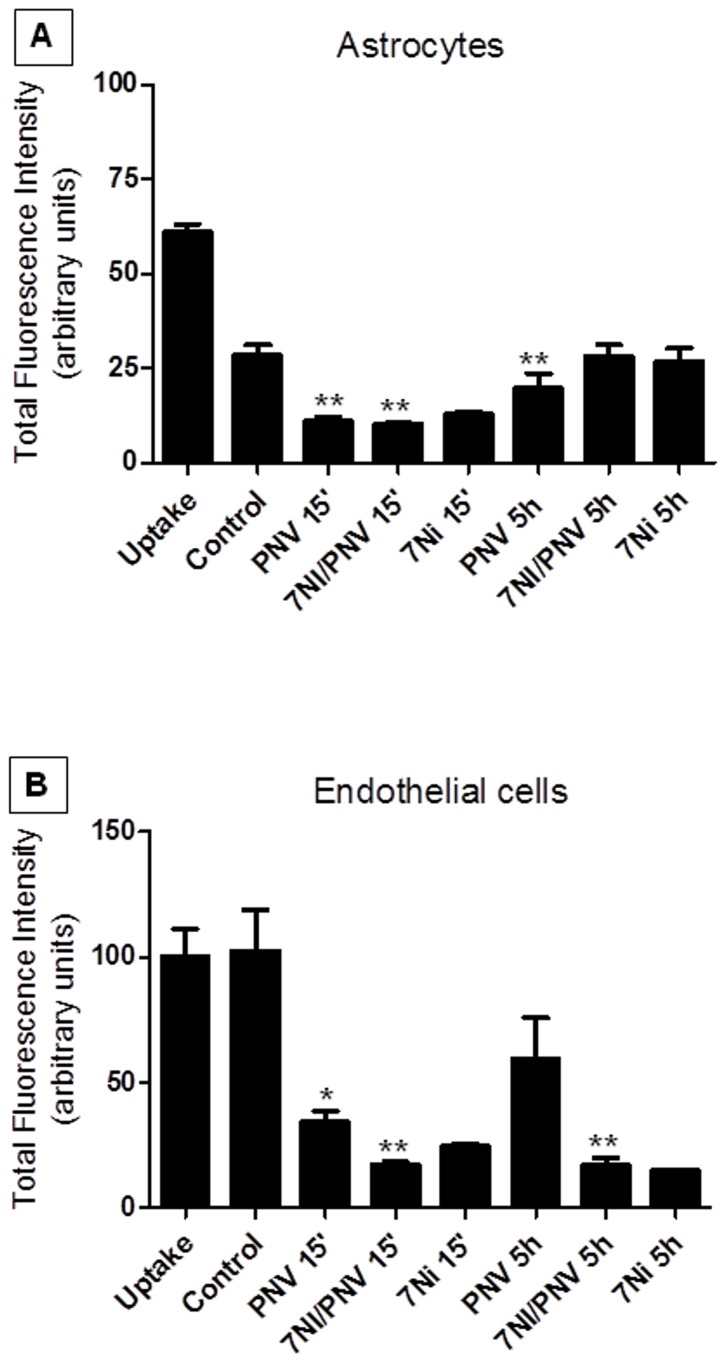
MRP1 activity in astrocytes (A) and endothelial cells HUVEC (B). The activity of MRP1 was assessed by flow cytometry through the fluorescein substrate. The values correspond to the percentage of fluorescence intensity into the cells of the Uptake sample, the maximum fluorescence obtained, and were expressed in Relative Fluorescence Units. In astrocytes, PNV induced immediate (15 min after exposure) decrease in the cellular fluorescence, indicating that MRP1 efflux activity was improved, compared with control; 5 h following envenoming, cells maintained MRP1 activity, although it was not as intense as at 15 min. 7NI+PNV treatment (for 15 min) also induced MRP1 activity in astrocytes, however this treatment for 5 h stopped efflux activity. 7NI *per se* (both at 15 min or 5 h) did not induce efflux activity. In endothelial cells, PNV induced a decrease in fluorescence at 15 min time-point, but not at 5 h. 7NI+PNV induced more significant MRP1 activity both at 15 min and 5 h in endothelial cells. 7NI treatment *per se* (used as a control of 7NI/PNV) showed no change in the MRP1 activity relative to 7NI/PNV. The values correspond to the values of three independent experiments. *p<0.05, **p<0.01, ***p<0.001, compared to control cells. Data was analyzed by one-way ANOVA, followed by the Tukey-Kramer post-test.

Fluorescence intensity was decreased significantly at 15 min comparing with DMEM-treated cells (control), suggesting efflux activity. At 5 h, the fluorescein efflux remained significant relative to control, but removal had decreased relative to PNV-15 min, indicating time-dependent MRP1 efflux activity (Figure 1A).

#### *7NI/PNV-treated astrocytes*

The fluorescence intensity was decreased significantly at 15 min relative to control (DMEM-treated) indicating fluorescein removal, whereas there was no difference relative to fluorescein removal at 5 h, thus indicating that the effect of nNOS-derived NO on the MRP1-efflux activity is time-dependent in isolated astrocytes (Figure 1A).

#### *PNV-treated HUVEC*

Fluorescence was significantly lower at 15 min after PNV exposure compared to control cells, but at 5 h the removal of fluorescein had diminished, thus indicating no significant difference relative to DMEM-treated HUVEC. This suggests that PNV treatment affects MRP1 activity time-dependently (Figure 1B).

#### *7NI/PNV-treated HUVEC*

Relative to the DMEM control, MRP1 efflux activity decreased significantly at 15 min post-treatment; the decrease was greater than in cells treated with PNV alone, thus indicating that the removal of fluorescein was potentiated in the absence of nNOS-derived NO. Interestingly, cells where removal activity had stopped after 5 h of treatment with PNV alone, when treated with 7NI+PNV for 5 h resulted in a significant decrease of fluorescence intensity, indicating reactivation of MRP1 activity (Figure 1B). The findings seem to indicate that NO signaling re-activates efflux exhaustion of MRP1.

#### *7NI-treated astrocytes and HUVEC*

7NI alone, used as control, did not show significant differences in the MRP1 efflux activity when compared with that of 7NI/PNV, in two tested periods (15 min and 5 h), in both astrocytes and HUVEC cells.

### In vitro experiments: Quantitative Real Time-PCR (qRT-PCR) analysis

#### *mrp1*

**Figure 2:**
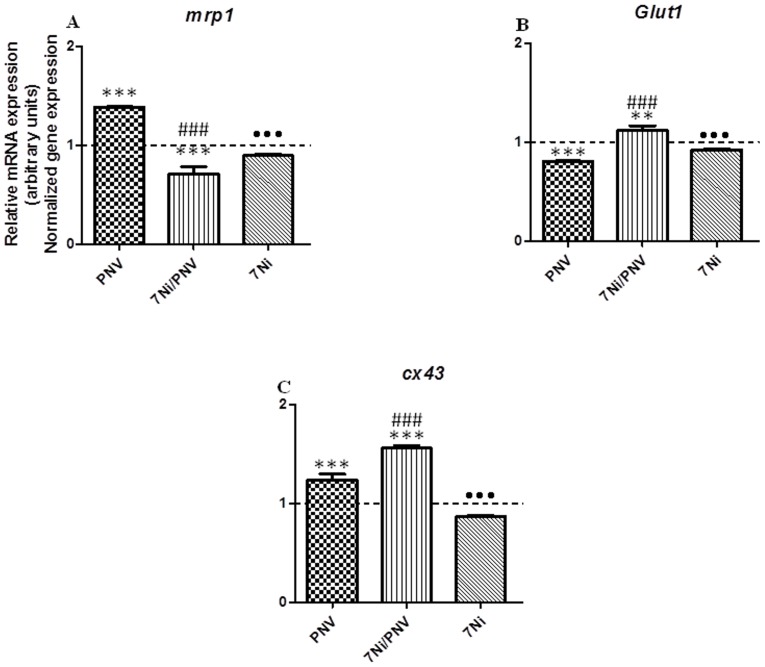
Effect of PNV and involvement of neuronal nitric oxide synthase in expression of mrp1, glut1 and cx43 genes, in astrocyte primary culture, by Real Time PCR. PNV increased mRNA levels of *mrp1* and nNOS blockade by 7NI counteracted this effect. *glut1* mRNA was decreased by PNV, but 7NI+PNV treatment also neutralized this PNV effect. However, *cx43* mRNA level was increased by PNV and even more by 7NI plus PNV. In 7NI *per se* treated-astrocytes *mrp1* gene expression was significantly above the level found in cell treated with 7NI/PNV; in contrast, *glut1* and *cx43* gene expression of astrocytes treated with 7NI alone was significantly below the level found in 7NI/PNV-treated cells. Fold change in genes expression was analyzed by the 2^−ΔΔCT^ method. Graphs showed relative gene expression, considering control as 1 (dotted line in graphs). Data is mean ± S.D.M. (**p<0.01 and ***p<0.001 *vs*. Control; ###p<0.001 *vs*. PNV; •••p<0.001 *vs*. 7NI/PNV).

PNV-treated astrocytes showed a 38% increase of *mrp*1 mRNA level at 2 h. Treatment with 7NI plus PNV significantly counteracted the effects of PNV leading to a 67% reduction below the level found in PNV-treated cells and a 29% reduction relative to baseline (Figure 2A). In the 7NI-treated astrocytes *mrp1* gene expression was significantly above the level found in cells treated with 7NI/PNV, but significantly below that found in PNV-treated cells.

#### *glut1*

PNV decreased *glut1* mRNA by 20% below baseline in cultured astrocytes. The inhibition of nNOS (7NI/PNV-treated astrocytes) reversed this effect by promoting a 12% increase above baseline and a 32% increase compared to PNV-treated cells (Figure 2B). 7NI *per se* decreased *glut1* gene expression in relation to 7NI/PNV treatment; however, the decrease was minor than the found in PNV-treated cells.

#### *cx43*

Two hours of PNV exposure induced a 25% increase in *cx43* mRNA in astrocytes, compared to the physiological level of untreated cells. Co-exposure of 7NI + PNV elevated the mRNA expression to 56% above baseline and 26% above the level found in PNV-treated astrocytes (Figure 2C). Incubation with 7NI alone significantly decreased gene expression compared with 7NI/PNV treated cells.

### In vivo experiments: WB data

#### MRP1

While hippocampal MRP1 level increased by 70% in PNV-treated rats at 5 h, in the cerebellum the protein level remained unchanged at all post-envenoming time-points (Figure 1A, B, C).

#### GLUT1

**Figure 3:**
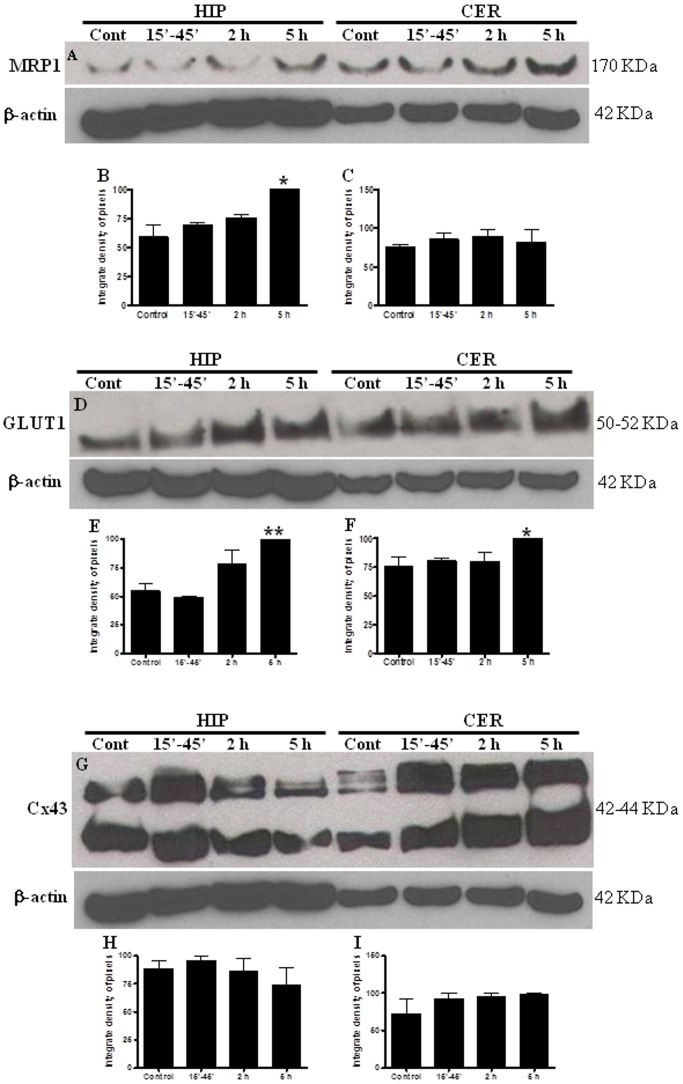
Western blotting for MRP1, GLUT1 and Cx43 in cerebellum (CER) and hippocampus (HIP). The time course of PNV treatment (15–45′, 2 and 5 h) was compared with control group. MRP1 expression was increased 5 h after envenoming in hippocampus. GLUT1 expression increased in PNV-5 h time-point, in both regions. No significant difference was observed in Cx43 expression. Values represent the mean ± S.E.M. The results were confirmed in three sets of experiments. *p<0.05, **p<0.01.

At 5 h following treatment of rats with PNV increases of 81% and 32% were observed in the hippocampal and cerebellar GLUT1, respectively, relative to saline-treated rats (Figure 3D, E, F).

#### Cx43

The total level of the main gap junction-forming protein remained unchanged in the hippocampus and cerebellum throughout the time-points post-PNV envenoming (Figure 3G, H, I).

### In vivo experiments: IF data

#### MRP1

**Figure 4:**
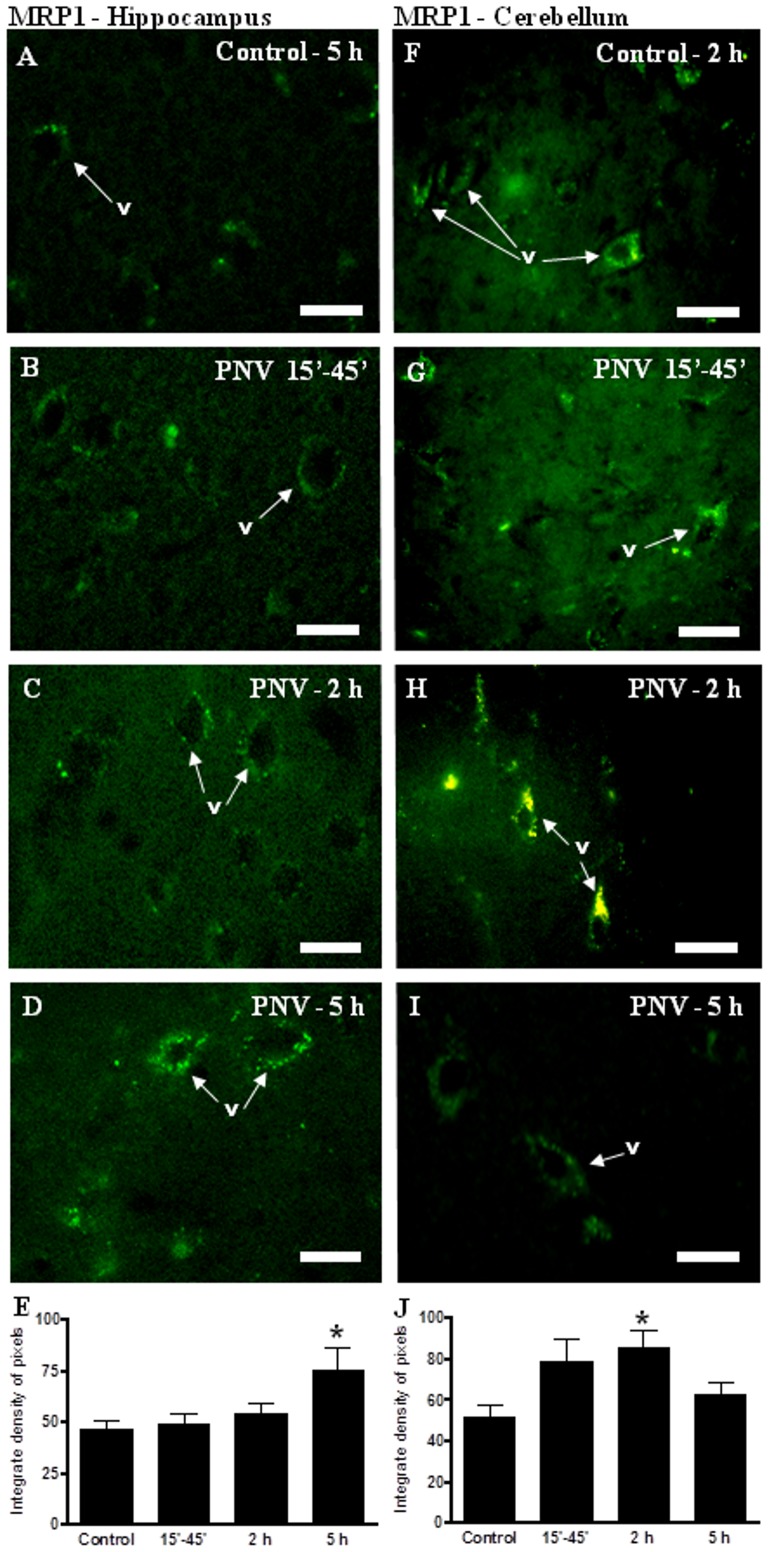
Immunofluorescence (IF) for MRP1 in hippocampus (A–D) and cerebellum (F–I). MRP1 labeling was increased in hippocampus after 5 h of envenoming. In the cerebellum, PNV-2 h showed an increase in MRP1. E and J show IF densitometric values for the hippocampus and cerebellum, respectively (*p<0.05 relative to control). Values represent the mean ± S.E.M. The results were confirmed in three sets of experiments. v =  blood vessel. N = 5 per group. Bar  = 35 µm.

MRP1 immunolabeling appeared as fluorescent dots outlining the endothelial wall of the hippocampal and cerebellar microvessels. At 5 h, PNV-treated animals showed a 62% increase in the MRP1 expression in hippocampus and a 66% increase at 2 h in the cerebellum in relation to respective control group (Figure 4A–J).

#### GLUT1

**Figure 5:**
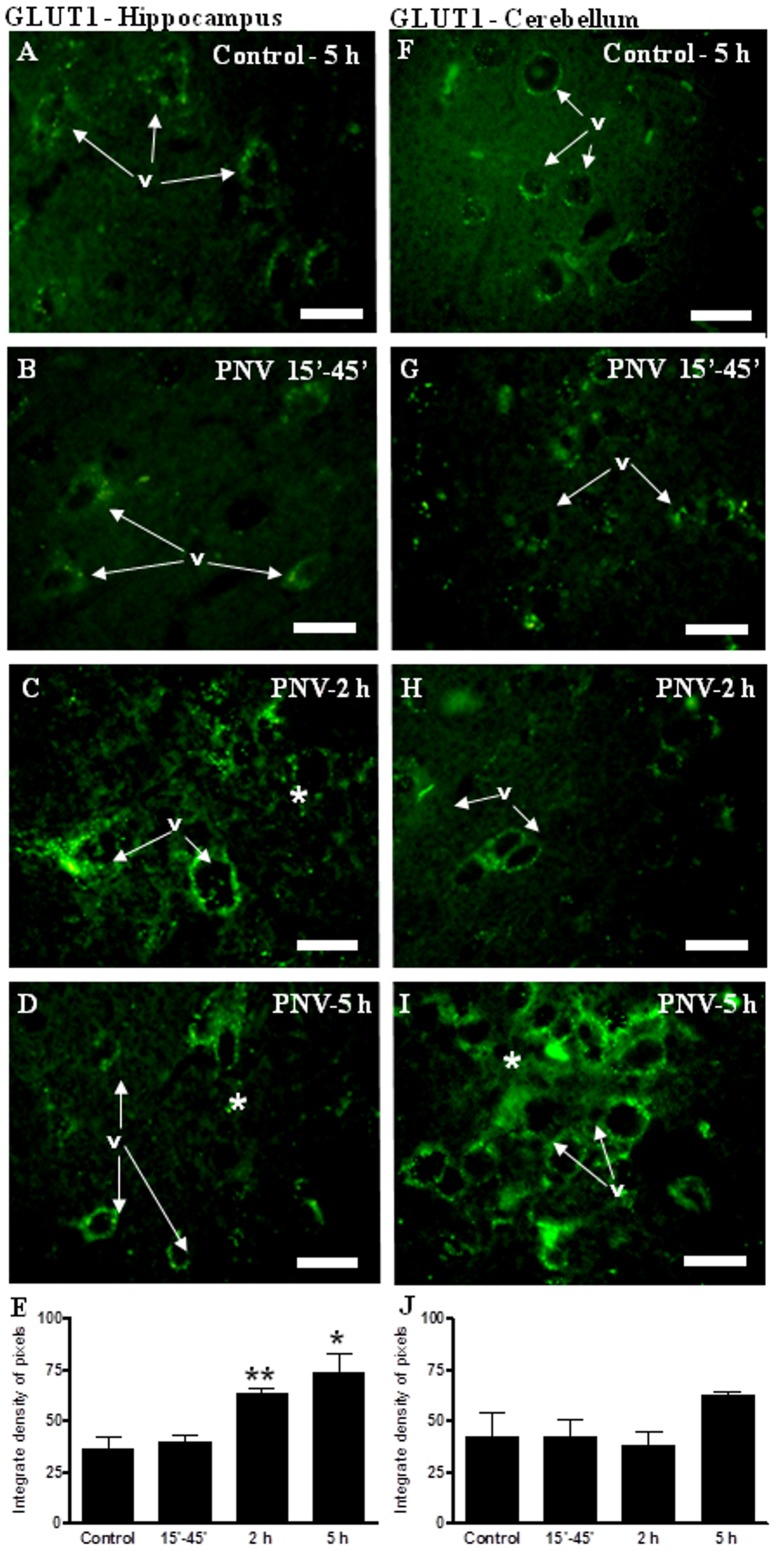
Immunofluorescence (IF) for GLUT1 in hippocampus (A–D), and cerebellum (F–I). In hippocampus, GLUT1 immunolabeling increased 2 and 5 hours after envenoming. In the cerebellum, no significant changes were observed. E and J show the IF densitometric values for the hippocampus and cerebellum, respectively (*p<0.05, **p<0.01 relative to control). Values represent the mean ± S.E.M. The results were confirmed in three sets of experiments. v =  blood vessel, * =  labeled parenchyma. N = 5 per group. Bar  = 35 µm.

Anti-GLUT1 immunolabeling was tenuous and discontinuous around vessels in controls; PNV promoted 80% (2 h) and 102% (5 h) significant increases in anti-GLUT1 reactivity around vessels and across the parenchyma, mainly in the hippocampus (see ^(^*^)^). In the cerebellum of PNV-treated rats, anti-GLUT1 was increased at 5 h, compared to control (Figure 5A–J).

#### Cx43

**Figure 6:**
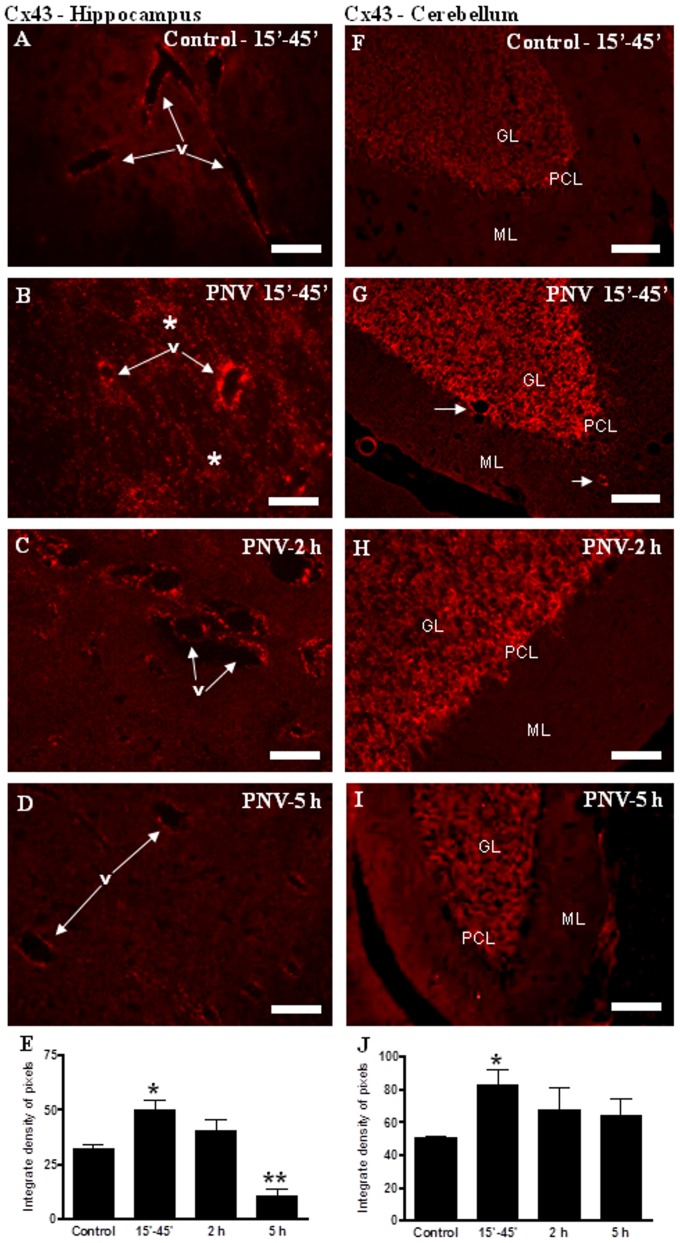
Immunofluorescence (IF) for Cx43 in hippocampus (A–D) and cerebellum (F–I). Cx43 expression was increased after 15–45′ in the hippocampus and cerebellum. In the hippocampus, there was an intense decrease after 5 h of envenoming. E and J show the IF densitometric values for hippocampus and cerebellum, respectively (*p<0.05, **p<0.01 compared to control). Values represent the mean ± S.E.M. The results were confirmed in three sets of experiments. v =  blood vessel; * =  labeled parenchyma; GL – glomerular layer; ML – molecular layer; PCL  =  Purkinje cell layer. Bars  = 70 µm (A–D, F, G, I); 35 µm (H).

In the control group, Cx43 immunoreactivity outlined microvessels in the hippocampus and cerebellum. This was faint in the Purkinje and granular layers (Figure 6A and 6F). PNV treatment induced an immediate enhancement of labeling in both layers followed by a gradual decrease (Figure 6B–D and 6G–I). Comparing with the control group, the quantification of pixel density (Figure 6E,J) showed a 56% increase in the hippocampus and a 64% increase in the cerebellum in anti-Cx43 staining, followed by a 200% decrease below baseline (5 h) in the hippocampus only.

#### Cx43 and GFAP

**Figure 7:**
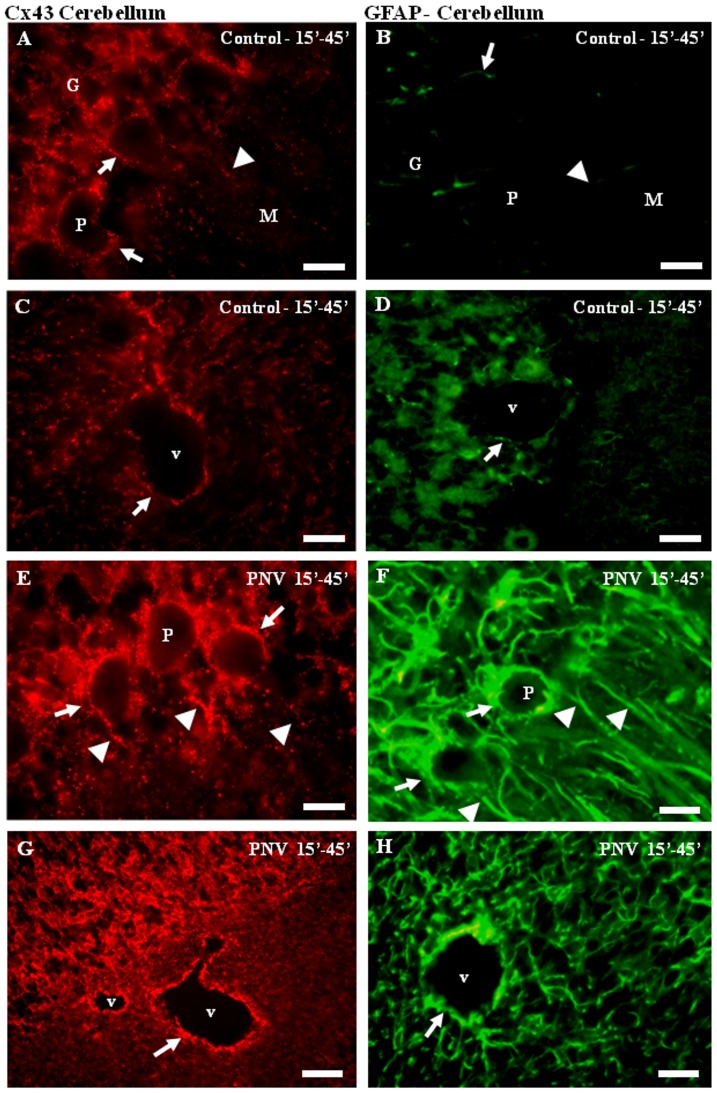
Cx43 and GFAP imunofluorescence in sections of cerebellum. A, C, E, G represent Cx43 and B, D, F, H represent GFAP immunolabeling. A–D showed control group and E–H showed PNV-15-45′ group. There is a correlation between Cx43 and GFAP labeling (arrows and arrowheads). This indicated that Cx43 are positive mainly in astrocytes. G =  Granular layer, P =  Purkinje layer, M =  Molecular layer, v =  blood vessel. Bar  = 70 µm for all panels.

In order to show that increases of Cx43 level paralleled with increases of GFAP in PNV-treated rats, frozen sections of the cerebellum (Figure 7) and hippocampus (not shown) were immunostained for GFAP and Cx43. Compared to controls (panels A, B, C, D), animals exposed to PNV showed an immediate (at 15–45 min) increase in the expression of Cx43 (see also IF and WB data) and induced GFAP expression in reactive astrocytes (panels E, F, G, H). Co-localization of Cx43 and GFAP was intense around Purkinje cells, granule neurons, the wall of vessels, and in astrocytic processes within the molecular layer. As expected, while Cx43 labeling appeared as serial dots, GFAP labeling was continuous, since gap junctions form isolated communicating channels in the plasma membrane, while GFAP is part of the intermediate filaments of the glial cytoskeleton distributed along the astrocyte processes and perikaryum. In the hippocampus, GFAP and Cx43 labeling increased around blood vessels and throughout the hippocampal parenchyma (not shown).

## Discussion

The results of the present study found that *P. nigriventer* armed-spider venom is a substrate for MRP1 activity in endothelial and astrocyte cells. PNV also affected the expression of *mrp1*, *glut1* and *cx43* mRNAs transcripts in astrocytes. Furthermore, evidence suggests that NO derived from nNOS could synergistically and/or antagonistically mediate the PNV effects. *In vivo*, the venom alters the translational expression of MRP1, GLUT1 and Cx43 proteins in the microvessel wall. Cx43 upregulation was detected in reactive astrocytes of the cerebellum and hippocampus and outlining the Purkinje neurons. All of these effects were time- and cell/region-modulated. To the best of the authors' knowledge this is the first study to show alterations of these proteins by spider venom that disrupt the BBB, and that is also known to affect other neurovascular systems [13].

The present immunohistochemistry-based *in vivo* MRP1 labeling is in conformity with its strategic location at the ECs of brain microvessels [18] and end-feet processes of astrocytes [19], [20]. *In vitro*, the ready increase in MRP1 activity in ECs and astrocytes as early as 15 min after venom exposure coincides with the onset of signs of serious intoxication of the animals. The increase in MRP1 efflux activity suggests a rapid induction of a pro-homeostasis protective mechanism. However, 51h later, endothelial cells no longer showed efflux activity while astrocytes showed activity deceleration in comparison with that seen at 15 min. Clinically, at this time-point the animals showed signs indicating that recovery of intoxication is underway [6], [21]. The absence of MRP1 activity in ECs and its slowdown in astrocytes at 5 h may imply that an appreciable amount of venom has been pumped out or that the efflux mechanism has undergone interference of second messengers, or both.

One such candidate is nitric oxide, a second messenger already known to affect transport at the blood-brain interface [22]–[24]. MRP1 belongs to the ATP-driven efflux pump family of transmembrane multidrug resistance-associated proteins (MDR) which mediate the removal of cytotoxic substances and drugs from the CNS. MRP1 acts as a glutathione S-conjugate efflux pump (GS-X pump) by transporting organic anions and several drugs which are conjugated with glutathione (GCH) [25]. The present findings found that nNOS-derived NO could be one of the mediators of the PNV effect at the BBB, and that the mediation is modulated according to the type of cells and time of venom exposure. The involvement of the nNOS/NO system has been already described to mediate the cavernosal relaxation induced by PnTx2-6, a toxin isolated from PNV [13], [26]. Therein, the toxin neurovascular effect was potentiated by NO produced by nNOS.

In the present study, the co-treatment of ECs with PNV plus the selective nNOS inhibitor (7NI) not only promoted enhancement of efflux activity at 15 min but reactivated pump activity at 5 h. The improvement of pump activity in the absence of nNOS-derived NO suggests that at that time-point (5 h) the NO dampens efflux activity, so working antagonistically to the efficiency of toxicant removal in ECs. Conversely, on the glial cells, the effect of NO seems to contribute positively to the removal of the toxicant, since MRP1 efflux activity in astrocytes treated with 7NI + PNV, which was in process in cells incubated with PNV alone, stopped efflux activity in the absence of NO produced by nNOS. Such effect was time-dependent, as it was only found at 5 h. At 15 min, efflux activity was maintained in 7NI/PNV-treated astrocytes equal to that found in PNV-treated cells. The findings suggest that NO intervention in MRP1 activity can be temporally diverse and can depend on cell-type. The results also show that astrocytes possess a more efficient mechanism for substrate (PNV) extrusion than the peripheral ECs used herein. Available literature indicates that the amount of MRP1 is greater in astrocytes than in the brain ECs of rats [27], [28] leading to the conclusion that astrocytes possess an efficient mechanism against drug (PNV) accumulation.

Besides NO, other mechanisms could affect the temporal-related MRP1 activity changes. The possibility that the pump could be rendered defective by the toxic accumulation of PNV inside the astrocytes or by failure in the conjugation of GSH, or another reason, cannot be discarded. In fact, relevant evidence shows that the transporter activity of MRP1 can be caused by endogenous-induced alterations in the glutathione metabolism [29]–[31]. Relevant literature reports links between MRP1 drug resistances vs. GSH cellular levels vs. drug-metabolizing cytochrome P-450 (CYP) induction in response to xenobiotics. CYP enzymes encompass a super-family of hemoproteins that play a role in the biotransformation of a wide range of endogenous and exogenous compounds [32]. CYP induction in response to xenobiotics occurs concomitantly with the generation of free radicals and depletion of intracellular GSH content [29], [30]. This hinders MRP1 activity as it requires conjugation or co-transportation of drugs and organic ions with GSH. The presence of the drug-metabolizing cytochrome P450 in cultured endothelial cells and primary astrocytes [29], [33] and in the wall of blood vessels, astrocytes of the cerebellum and pyramidal neurons of all hippocampal subfields [34], [35] evidences the existence of endogenous alterations in GSH levels, and hence in MRP1 activity.

The inhibition of MRP1 activity has been also reported to occur through changes in the conformation of the MRP1 molecule [25], [36]. However, this did not seem to be the case in the present study, the data of which indicates that *mrp1* gene activity remained active, since *mrp1* mRNA increased in cultured astrocytes treated with PNV (Figure 2). In agreement, the translational event was not affected, since PNV-treated rats showed increased expression of MRP1 in the hippocampus and cerebellum (Figure 3 and 4). Altogether, the data cast doubt on the capacity of MRP1 to make conjugates and export them from PNV-treated cells. Further studies are necessary to clarify this, and to verify GSH stores inside the cells.

In relation to mediation by NO, it was noted that at the transcription level NO acted proactively in relation to MRP1 transcription, since in the absence of NO-derived nNOS (7NI+PNV-treated astrocytes) mRNA expression decreased significantly relative to both control and PNV-treated astrocytes. The data suggests that the gas acts synergistically with PNV promoting the increase of *mrp1* mRNA transcripts. This is worthy of note because in relation to MRP1 activity, NO inhibits the extrusion activity of the protein.

The complexity of NO mediation was also seen in relation to the influx transporter, GLUT1. 7NI+PNV treatment increased *glut1* gene expression which had been reduced by PNV alone. The finding reveals that NO produced by nNOS may have had a critical role in a possible reduction of the facilitative glucose transporter promoted by the spider venom and hence in a supposed lower supply of energy fuel from astrocytes for brain metabolism [37]. Nevertheless, the hippocampus and cerebellum of rats treated with PNV showed increased expression of GLUT1 indicating that translational events were stimulated by the venom. Such apparent discrepancy may be attributed to *in vitro* versus *in vivo* experiments and different time scales, but also to sometimes divergent regulatory mechanisms acting at the translational stage and respective coding gene [38].

Little has been reported about interaction between multidrug resistance protein and glucose transporter protein. However, it is conceivable that cytotoxic insult to the CNS would demand greater resources of glucose and a mechanism for eliminating the toxic agent. In fact, studies in *in vitro* cell lines have shown an increased rate of facilitative glucose transport and level of GLUT1 expression in parallel with increased vincristine resistance, active vincristine efflux and decreased vincristine steady-state accumulation. Moreover, glucose transport inhibitors (cytochalasin B and phloretin) block the active efflux and increase steady-state accumulation of vincristine [39]. Contrastingly, increased expression/content of GLUT1 reflects elevated levels of circulating glucose, and the increase of brain glucose utilization has been correlated positively with elevated expression of GLUT1 and vice-versa [40]. It may be suggested that significant increases of GLUT1 in the hippocampus and cerebellum of PNV-treated rats could be evidence of enhanced glucose transport and probable high energy metabolism demand after envenomation. The significant increase of *glut*1 transcripts after the inhibition of nNOS revealed that NO mediation is synergic with PNV against facilitative glucose transport. The results of the present study indicate that PNV is a useful tool for research into MDR phenotype development, drug efflux and glucose uptake mechanisms.

PNV increased *cx43* gene expression, an effect that was potentiated by the absence of NO produced by nNOS. The increases in the *cx43* transcripts suggest increased preparation for translational events, with NO antagonistically mediating the effects of PNV. The increase in *cx43* gene activity *in vitro* paralleled increases in Cx43 protein expression, though the latter was only observed through the density of pixels of anti-Cx43 labeling (IF data) but not in the total amount of protein in the hippocampus and cerebellum (WB data). The lack of significance obtained from WB analysis indicates that the formation of new intercellular channels is not uniform throughout the cerebellum and hippocampus parenchyma as previously reported [41]. In fact, the increase of Cx43 immunoreactivity was marked around blood vessels and around Purkinje and granule neurons but less expressive in the molecular layer of the cerebellum (Figure 7). Such increases in Cx43 expression occurred 15 to 45 min after envenomation when animals already manifested signs of intense intoxication [4], [8], [21]. At subsequent time-points, Cx43 staining underwent a major reduction in the hippocampus and showed a tendency for reduction in the cerebellum (Figure 6), coincidently with amelioration of the toxic condition of the animals [4], [8], [21]. The increase of Cx43 protein expression implies the establishment of intercellular channels for diffusion of chemical and electrical information between the reactive (GFAP+) astrocytes [42]. Connexin 43-formed channels are strictly linked to modifications of ionic composition of extracellular CNS compartment, with calcium oscillations possessing a critical role [43]. PNV contains excitotoxic neuropeptides [11] and PnTx1-3, a neurotoxin isolated from PNV, has been shown to increase the frequency of Ca^2+^ oscillations in *in vitro* GH3 cells [44]. The significant further reduction of Cx43 expression, especially in the hippocampus (5 h post-PNV), could be a mechanism for avoiding cell damage [45], since PNV induces FOS induction (2 h post-PNV) in neurons [6] and the decrease of gap junctions (GJs) formation/communication in astrocytes could be controlled by neurons [42].

A further interesting point is the relationship between Cx43 and glucose metabolism. Molecular pathways suggest a potential link between GJs and energy metabolism in astrocytes. Studies have shown that the inhibition of Cx43 increases glucose transport by astrocytes through GJs [46] and up-regulation of GLUT1 [47], which is then associated with up-regulation of Na^+^/K^+^-ATPase activity [48]. The basis of this relationship is still unknown, but could be related to the blocking effect of PNV on K^+^ channels [11]. The data indicates that PNV is instrumental for studies related to the interaction of Cx43, GLUT1 and MRP1.

In conclusion, the present study shows that the PNV is a substrate for MRP1 activity. Also, the study provides evidence that nNOS-derived NO is involved in the mediation of PNV effects, apparently through a complex dual mediation at the BBB level: as an enhancer of *mrp1* transcription in astrocytes and as an inhibitor of MRP1 efflux activity in endothelial cells. Data showing differences between the type of cells and between the hippocampus and cerebellum, two regions in which PNV disrupts the BBB, is interesting as it allows the detection of regional differences in energy metabolism, capacity for drug exclusion/distribution and communication between cells in PNV-intoxicated rats. The study not only provides information for therapeutic purposes in relation to victims of *Phoneutria nigriventer* envenomation, but also reveals the potential natural resources that exist in venomous fauna (particularly rich in Brazil) in terms of pharmacologically active components.
